# Autophagy-Related ncRNAs in Pancreatic Cancer

**DOI:** 10.3390/ph15121547

**Published:** 2022-12-13

**Authors:** Simone Donati, Cinzia Aurilia, Gaia Palmini, Irene Falsetti, Teresa Iantomasi, Maria Luisa Brandi

**Affiliations:** 1 Department of Experimental and Clinical Biomedical Sciences, University of Florence, Viale Pieraccini 6, 50139 Florence, Italy; 2 Fondazione Italiana Ricerca sulle Malattie dell’Osso (FIRMO Onlus), 50141 Florence, Italy

**Keywords:** pancreatic tumors, autophagy, non-coding RNAs, miRNAs, long non-coding RNAs, circular RNAs

## Abstract

Pancreatic cancer (PC) is a malignancy accounting for only 3% of total cancers, but with a low 5-year relative survival rate. Approximately 80% of PC patients are diagnosed at a late stage when the disease has already spread from the primary site. Despite advances in PC treatment, there is an urgently needed for the identification of novel therapeutic strategies for PC, particularly for patients who cannot undergo classical surgery. Autophagy is an evolutionarily conserved process used by cells to adapt to metabolic stress via the degrading or recycling of damaged or unnecessary organelles and cellular components. This process is elevated in PC and, thus, it contributes to the onset, progression, and cancer cell resistance to chemotherapy in pancreatic tumors. Autophagy inhibition has been shown to lead to cancer regression and to increase the sensitivity of pancreatic cells to radiation and chemotherapy. Emerging studies have focused on the roles of non-coding RNAs (ncRNAs), such as miRNAs, long non-coding RNAs, and circular RNAs, in PC development and progression. Furthermore, ncRNAs have been reported as crucial regulators of many biological processes, including autophagy, suggesting that ncRNA-based autophagy targeting methods could be promising novel molecular approaches for specifically reducing autophagic flux, thus improving the management of PC patients. In this review, we briefly summarize the existing studies regarding the role and the regulatory mechanisms of autophagy-related ncRNAs in the context of this cancer.

## 1. Introduction

The pancreas is a glandular organ that derives from the foregut endodermal germ layer with both exocrine and endocrine functions [1,2]. This gland is located in the retroperitoneal space of the upper abdomen, and it functions as an exocrine gland producing enzymes that are crucial to digest proteins (i.e., trypsin and chymotrypsin), carbohydrates (i.e., amylase) and lipids (i.e., lipase) contained in food, and as an endocrine gland producing hormones (i.e., insulin, somatostatin, and glucagon) responsible for the maintaining of blood sugar levels in a normal range [3,4,5].

Pancreas malfunctions could lead to the development of common disorders, such as diabetes, pancreatitis, and malignant neoplasm of the pancreas [6,7,8].

Pancreatic ductal cell adenocarcinoma (PDAC) is the most common subtype of exocrine cancer and represents approximately 90% of pancreatic malignancies, while tumors derived from endocrine pancreatic islet cells are relatively uncommon and constitute 5% of the pancreatic neoplasms [9,10].

Early and accurate detection of pancreatic cancer (PC) are the most important challenges in the primary care of these tumors because they may help doctors to plan the specific treatment option for patients [11]. Diagnosis is usually based on a combination of a set of imaging techniques, such as endoscopic ultrasonography (EUS), magnetic resonance imaging (MRI), and computed tomography (CT), the measurement of serological tumor markers (i.e., carbohydrate antigen 19-9 (CA19-9), osteopontin (OPN), carcinoembryonic antigen (CEA), S100A6, and macrophage inhibitory cytokine 1 (MIC-1)), the appearance of clinical features, and the “gold standard” histological examination [12,13,14].

In recent years, a significant breakthrough with respect to morphological changes and key genetic factors has been made for defining PC, even though the exact causes of PC are still not clear. Some risk factors, such as age, sex, ethnicity/race, as well as other modifying factors, including obesity, smoking, occupational risk factors, and diabetes, which could increase the potential for acquiring genetic mutations, have been associated with increased susceptibility to PC development [15]. Additional studies demonstrated that the presence of *Helicobacter pylori* infection [16], chronic hepatitis B [17], and hepatitis C [18] correlate in a positive way with the risk of PC, although the mechanisms underlying this association are not well understood so far.

The lack of symptoms is the main reason for delayed diagnosis and the treatment of PC. The occurrence of clinical manifestations commonly indicates an advanced stage, and the most common presentations are abdominal pain, progressive weight loss, nausea, vomiting, anorexia, and jaundice [19].

For this reason, PCs are notoriously difficult to treat; usually, the treatment plans for these tumors are based on the possibility of surgical intervention to resect the carcinogenic mass [12]. Most pancreatic cancers (PCs) are only diagnosed after they have already spread beyond the pancreas and therefore patients are not eligible for surgery [20,21]. In fact, only 20% of patients are treated surgically for pancreatic tumors through a surgical procedure named Whipple (pancreatoduodenectomy), which involves the removal of the head of the pancreas, as well as part of the duodenum, gallbladder, and the common bile duct [22,23,24,25]. When cancer involves the body and tail of the pancreas, distal/subtotal pancreatectomy is advised [24].

For those patients with unresectable PC, chemotherapy is widely used, such as gemcitabine, oxaliplatin, modified leucovorin, 5-fluorouracil, and irinotecan [12]. Unfortunately, PC is characterized by an excessively dense desmoplastic reaction, which promotes chemotherapy resistance [26]. However, despite the limited efficacy in patients with advanced disease, chemotherapy plays a crucial role in the adjuvant setting inducing a partial response and alleviating symptoms in those individuals with metastatic PC.

In addition, for unresectable PC, little evidence exists now to support the effectiveness of radiotherapy. However, radiotherapy could be used as a palliative care option for those patients with unresectable locally advanced tumors to kill cancer cells for preventing tumor growth and recurrence [27].

Given the limited benefits of approved therapies in PC, immunotherapy is an effective therapeutic strategy for a wide range of diseases and, therefore, it has been recently considered as a novel non-chemotherapeutic approach for PC patients [28,29]. The goal of immunotherapy is to induce the immune system response against cancer, by improving the recognition of tumor cells by the host immune system [30]. Nevertheless, only the programmed cell death 1 (PD-1) immune checkpoint inhibitor (ICI) pembrolizumab received Food and Drug Administration (FDA) authorization [29]. Since 2018, when the American Society of Clinical Oncology (ASCO) updated the clinical practice guideline for metastatic pancreatic lesions, this monoclonal antibody targeting PD-1 has been recommended as standard-care second-line treatment in patients with microsatellite instability (MSI) or DNA mismatch repair deficiency (dMMR) [31]. However, the low incidence of MSI in PC patients (<1%), makes that not all patients are eligible for ICI-based treatments [32,33]. Moreover, it is well established that the immunosuppressive tumor microenvironment and an intense desmoplastic reaction render pancreatic cells capable of evading immune surveillance, resulting in the failure of immunotherapy-based strategies when applied to PC patients [34].

In this light, there is an urgent need to develop novel promising diagnostic and therapeutic approaches for improving PC patients’ treatment outcomes.

Autophagy is a central highly evolutionary conserved homoeostatic mechanism able to maintain the stability of the microenvironment inside cells, which in recent years has been reported to be involved in cancer pathogenesis and drug resistance [35,36]. Under normal conditions, autophagy takes place at a constant, basal level, whereas it appears overactivated in related-stress circumstances, such as nutrient starvation, DNA damage, and hypoxia [37]. This multi-step process is regulated by highly conserved genes, named autophagy-related genes (ATGs), and comprises five phases: (I) initiation, mediated by Unc-51-like kinase 1 (ULK1) complex (i.e., ULK1, ATG13, FIP200, and ATG101); (II) autophagosome nucleation, which involves the VPS34, Beclin 1 (BECN1), ATG14 L, and VPS15; (III) expansion and elongation of the autophagosome membrane, which requires the formation of the Atg12–Atg5-Atg16 complex and microtubule-associated protein 1 light chain 3 (LC3); (IV) closure and fusion with lysosomes to form the autolysosome; and (V) degradation of the inner cargos by hydrolytic enzymes [38,39].

Deregulation of the autophagy mechanism has been associated with the development, metabolism, and progression of PC, depending on several factors, including microenvironment, tumor stage, immunometabolic state, etc. [40,41,42]. Different studies highlighted that PDAC exhibits higher basal levels of autophagy and how this increase can be crucial for tumor survival and development. In fact, it has been observed that PDAC cells showed significantly higher levels of LC3 compared to normal pancreatic cells [41]. Regarding PC progression, it is supported by the excessive production of fibrotic stroma, and the presence of pancreatic stellate cells (PSCs), a collagen source [42,43]. In this light, it has been observed that autophagy inhibition is associated with higher PSCs in a quiescent state and reduced levels of the extracellular matrix and IL-6 production, thus decreasing the inflammatory response and tumor growth [44]. Recent evidence also showed that autophagy inhibition could suppress the pancreatic tumor’s progression by inducing DNA damage and apoptosis, and by suppressing tumor cell proliferation [45,46].

An expanding body of knowledge underlying the molecular pathogenesis of the disease has revealed that genetic alterations, such as *KRAS* mutations, and especially epigenetic abnormalities, are hallmarks of pancreatic tumors [4,47].

Epigenetic changes are a set of intracellular mechanisms that impact gene expressions independently of modifications of primary DNA sequences, which include DNA cytosine methylation, histone protein modifications, and the action of non-coding RNAs (ncRNAs) [48,49]. According to the number of nucleotide lengths, ncRNAs are classified into two subgroups: (1) a category of short ncRNAs with lengths fewer than 200 nucleotides (i.e., microRNAs (miRNAs), small interfering RNAs (siRNAs), Piwi-interacting RNAs (piRNAs), small nucleolar RNAs (snoRNAs), and small nuclear RNAs (snRNAs)); and (2) ncRNAs with lengths longer than 200 nucleotides, referred to as lncRNAs [50].

This latter category can be additionally divided into intragenic and intergenic lncRNAs, depending on their genomic positions [49,51,52,53,54]. Furthermore, it has also been reported that some subtypes of lncRNAs can self-circularize by linking their 3′ and 5′ ends with covalent bonds, thereby forming the class of circular RNAs (circRNAs) [52].

LncRNAs can function as competitive endogenous RNAs (ceRNAs), by competitively occupying the binding sequence of a target miRNA, thereby inhibiting its downstream action [49]. With regards to circRNAs, these circular molecules can either be translated to generate functional micropeptides or function as ceRNAs [55].

miRNAs are a class of small noncoding RNAs of approximately 18–25 nucleotides in lengths that negatively mediate the post-transcriptional regulation of gene expression [56,57]. The first miRNA, lin-4, was originally identified in *Caenorhabditis Elegans* (*C. Elegans*) in 1993 by Ambros and Ruvkun, followed by the identification of small RNAs in animals, plants, protists, and viruses (although not in bacteria) [58,59]. They account for only about 1% of the total human DNA, localizing predominantly in intergenic and intronic regions [60]. It has been estimated that miRNAs are involved in the regulation of at least 20–30% of human transcripts [61]. In most circumstances, miRNAs bind to the seed sequence (nucleotides 2–8) in the 3′ untranslated regions (3′-UTR) of the transcript target to inhibit their translation with imperfect complementarity and/or induce mRNA degradation via complete complementarity [62,63]. It has been reported that these small molecules can also interact with other gene regions, including the coding sequence, 5′-UTR, as well as promoter sequences, albeit more detailed studies are needed to characterize this latter interaction [64]. To date, there 2654 human miRNA sequences are listed in the miRbase Registry (available online: http://www.mirbase.org, accessed on 5 October 2022).

It is known that ncRNAs are involved in a wide range of biological processes, such as development, cell proliferation and differentiation, apoptosis and autophagy, and others [65,66,67,68]. The regulatory functions of miRNAs in the autophagic process were first discovered in 2009 when MIR30A was reported to regulate BECN1 expression, a critical autophagy-promoting gene [69]. Soon after this report, abnormalities in the autophagic-regulating miRNAs expression patterns have been associated with the pathogenesis of pancreatic tumors, suggesting them as novel potential biomarkers for diagnosis and are clinically important for the treatment of these tumors [70,71,72].

Reports suggest that lncRNAs are differentially expressed and involved in the progression of PC based on their impacts on the processes of cell proliferation, invasion, autophagy, apoptosis, and epithelial-mesenchymal transition (EMT) [73,74]. Regarding circRNAs, studies indicate that these molecules could be implicated in pancreatic tumor progression via accelerated autophagic response [75,76].

In this non-systematic review, we briefly provide an overview of autophagy-related miRNAs, lncRNAs, and circRNAs involved in pancreatic tumor pathogenesis, which could be used as a new promising approach for autophagy inhibition in PCs.

To achieve this purpose, we performed a literature search via PubMed/MEDLINE database by utilizing different combinations of appropriate terms, such as “pancreatic tumors”, “autophagy”, “non-coding RNAs”, “miRNAs”, “lncRNAs”, and “circRNAs”. All relevant studies were selected and reviewed.

## 2. Autophagy-Related miRNAs in PC

Although the gold standard for the treatment of pancreatic tumors is surgery, chemotherapy, and radiotherapy, these approaches are usually not effective due to the development of treatment resistance by cancer cells. Certainly, autophagy plays an important role in the mechanisms that cancer cells use to survive and grow. Indeed, it has been observed that increased autophagy flux improves cell survival resistance to treatments with radiation and chemotherapeutic agents [41,45,46]. Furthermore, in recent years, it has been interesting to observe that miRNAs can regulate autophagy by targeting different factors involved in this process, thus making these molecules possible candidate targets for the development of new therapeutic strategies against PC.

In this paragraph, we will review several studies that investigated the role of miRNAs in PC with a particular focus on their actions in the autophagic process.

Since the autophagy-related ncRNA mechanisms associated with PCs remain unclear, Wei et al. [77] explored potential ceRNA, miRNA, and mRNA pathways in PC-related autophagy via the construction of a ceRNA network. Initially, a microarray analysis revealed the differential expressions of 3.966 mRNAs, 3.184 lncRNAs, 9.420 circRNAs, and only two miRNAs (miR-154-3p and miR-663a-5p) in human PANC-1 cells treated with chloroquine diphosphate, an autophagic inhibitor. Using bioinformatics prediction tools, they identified a potential ceRNA network involved in the autophagy of PC cells composed of miR-663a-5p, 9 circRNAs, 8 lncRNAs, and 46 genes. Moreover, another ceRNA network consisting of miR-154-3p, 11 genes, 2 lncRNAs, and 5 circRNAs were also constructed. The two identified ceRNA could improve the understanding of autophagy in PC, paving the way for novel ideas and new directions for future research.

The purpose of a study by Zhang et al. [78] was to investigate the expression and function of miR-216a-5p in PC. Expression levels of miR-216a-5p were significantly downregulated both in PC tissues and cells, and plasma specimens of PC patients than their normal counterparts. Furthermore, miR-216a-5p levels were negatively correlated with PC peripancreatic lymphatic metastasis, advanced tumor, node, metastases (TNM) staging system, and perineural invasion. Functional studies revealed that miR-216a-5p inhibited PC cell growth and migration, either in vitro or in vivo. Bioinformatic analysis and a subsequent luciferase reporter assay established that miR-216a-5p binds and suppresses the expression of the translationally controlled tumor protein 1 (TPT1), an oncoprotein overexpressed in many human cancers that contributes to pancreatic tumor progression by regulating mTOR-dependent autophagy. Then, bioinformatics analysis, RNA pulldown, and expression studies confirmed that the reciprocal regulation of miR-216a-5p and LINC01133 plays a crucial role in PC tumorigenesis, suggesting both of these molecules as potential diagnostic biomarkers and therapeutic targets for PC.

In 2013, Wang et al. [79] obtained two radioresistant human pancreas cell lines (i.e., RR PANC1 and RR BxPC3) from their respective parental PANC1 and BxPC3 cells and performed a miRNA expression array, showing a different expression of 25 miRNAs in the former cells compared to the non-radioresistant ones. The subsequent analysis with the TaqMan quantitative Real-Time PCR (qPCR) confirmed that 4 miRNAs were downregulated, and 11 miRNAs were upregulated in the radioresistant cells compared to their parental cell lines. Both RR PANC1 and RR BxPC3 cells showed lower expressions of miR-23b and contemporary increased autophagy. Further analyses found that miR-23b was able to bind and inhibit the translation of autophagy-related gene 12 (ATG12) mRNA, an important regulator of autophagic flux. Subsequently, the experiments conducted in vitro and in vivo showed that the overexpression of miR-23b led to the suppression of autophagy and increased radiosensitivity. On the other hand, the use of chloroquine (CQ) did not show a synergistic effect with the increase of miR-23b; on the contrary, CQ appeared to increase the radiosensitivity of PC cells when the miR-23 expression was low, demonstrating the potential of miR-23b as a possible therapeutic target and prognostic marker for PC.

Zhang et al. [80] also attempted to study the role of miRNAs in the radiation resistance mechanism of PC cells. As described in the previous study, they found that radioresistant PC cells had reduced miR-216a expression and increased autophagy compared to control cells. In silico and in vitro analyses showed that miR-216a inhibited the production of Beclin-1 (BECN1), which is a crucial factor in the autophagy process, through its binding to the 3′-UTR region of BECN1 mRNA. Furthermore, it was observed that miR-216a overexpression in the cells caused increased apoptosis when exposed to radiation treatment. Finally, the strong expression of miR-216a in xenograft tumor models led to a reduction of the Beclin-1 expression and autophagy process with a consequent increase in cell sensitivity to irradiation treatment.

Given previous studies about the role of the transcription factor Yin-Yang 1 (YY1) in autophagy, Yang et al. [81] wanted to further investigate the function of this molecule in PC. After establishing that autophagy flux was higher in cancer cells than in healthy cells, they induced the overexpression and silencing of YY1 in PC cells and observed that higher expression of YY1 increased intracellular levels of LC3II and, thus, autophagy, whereas an opposite effect was observed when YY1 was downregulated, thus demonstrating its involvement in the autophagic process. Subsequently, it was seen that YY1 was able to bind the promoter and repress miR-30a transcription, which inhibited YY1, thus giving rise to a feedback mechanism, which regulates autophagic activity in PC cells.

To understand the effects of hypoxia on the autophagic process, Tian et al. [82] wanted to study the miRNAs differentially expressed in PC cells under normoxic and hypoxic conditions. They found that hypoxia-stimulated cells showed a particular reduction in the expression of miR-138-5p and that the overexpression was able to induce a substantial reduction in autophagy markers, including LC3-II and p62, as well as block serum starvation-induced autophagy flux. Then, it was shown that miR-138-5p did not exert its action on the autophagy-associated genes, such as ATG3, ATG5, and ATG7; however, it was able to interact and inhibit the translation of the silent mating type information regulation 2 homolog 1 (SIRT1) mRNA, which regulates the autophagy process by acetylating the Forkhead box protein O1 (FoxO1) and reducing the Rab7 protein levels. In addition, in vivo models showed that overexpression of miR-138-5p markedly slowed down tumor growth, decreasing both hypoxia-inducible factor 1-alpha (HIF-1α) and SIRT1 expression levels and, consequently, autophagy.

In 2018, Huang et al. [72] studied the role of miR-29c in the chemosensitivity of PC cells. First, they transfected Panc-1 cancer cells with an overexpression vector for miR-29c and observed that these cells showed increased sensitivity to gemcitabine treatment compared to the counterpart transfected with the NC (negative control) vector and an increased rate of the apoptotic process. Indeed, the Western blot assay showed that miR-29c increased the production of caspase 3 and Bax and decreased the expression of Bcl-2. Furthermore, it was revealed that miR-29c inhibited gemcitabine-induced autophagy and in parallel, downregulated ubiquitin-specific peptidase 22 (USP22), a deubiquitinating enzyme involved in autophagy induction. Subsequently, the miRanda algorithm identified complementary sequences between the 3′-UTR portion of USP22 and miR-29c, which was then confirmed by the luciferase reporter assay. In addition, the authors highlighted that miR-29c overexpression in a xenograft mouse model increased the effects of gemcitabine on tumor development precisely by negatively regulating USP22 expression and the autophagic process and by increasing apoptosis.

Instead, Wang et al. [83] investigated whether miR-137 could play a biological role in the chemosensitivity of PC cells. Initially, they observed that treatment of PANC-1 cells with doxorubicin (Dox) resulted in increased LC3-II/LC3-I and decreased p62 protein levels accompanied by a decrease in miR-137 expression, compared to untreated cells. Furthermore, the overexpression of this miRNA in dox-treated PANC-1 cells induced a substantial reduction in cell viability due to the increased sensitivity of the cells to the drug. In addition, an increased expression of miR-137 in PC cells had an inhibitor effect on dox-induced autophagy by targeting the ATG5 gene and by blocking autophagosome formation. This resulted in more cell sensitivity to dox and the rate of cell death. Furthermore, the experiments conducted in animal models showed the same results obtained in vitro, demonstrating that miR-137 behaved as an oncosuppressor, capable of inhibiting autophagy and making cancer cells more susceptible to chemotherapy.

The effects of the new oleic acid (OA) derivative, K73-03, on PC were investigated by Shopit et al. [84], which demonstrated the capacity of this compound to reduce the proliferation, migration, and colony formation of AsPC-1 PC cells in a dose- and time-dependent manner. The use of transmission electron microscopy (TEM) and then the Western blot method showed that K73-03 induced an increase in autophagy with a positive change in protein levels both of LC3 and BECN1. In addition, K73-03 was able to cause a dysfunction of mitochondrial activity by increasing the expression of apoptosis effectors, including cytochrome C, Bax, cleaved caspase-3, and cleaved PARP in the cytoplasm, inducing programmed cell death. At this point, they saw that K73-03 AsPC-1 cells showed a significant upregulation of miR-421 and downregulation of the serine protease inhibitor Kazal-type 1 (SPINK1) expression levels. In fact, it has been demonstrated that the OA derivative could reduce the expression of the transcription factor Zeste Cancer Homolog 2 (EZH2), which would silence the transcription of miR-421, which in turn may target the 3′-UTR of SPINK1. Thus, this signaling pathway could lead to an increase in autophagic flux and apoptosis. These findings were also obtained in in vivo experiments. Finally, K73-03 administration in SPINK1-overexpressing tumors has proved to be more effective than gemcitabine administration in reducing the SPINK1 expression and increasing autophagic and apoptotic processes.

Last year, Zhou et al. [85] illustrated that tetraspanin 1 (TSPAN1) could play a key role in the growth and progression of PC. Indeed, results obtained from the immunohistochemical analysis showed upregulated levels of TSPAN1 in PC patients compared to normal tissues. Furthermore, in vitro models of PC cells (i.e., MIA-PACA-1, SW1990, PANC-1, CAPAN-1, and ASPC-1) revealed that TSPAN1 expression was much higher compared to that present in the HPD E6-C7 of a normal pancreatic duct epithelial cell line, correlating with a poor prognosis of the disease. Furthermore, the MTT assay showed that TSPAN1 depletion was able to inhibit the proliferation of ASPC-1 and PANC-1 cell lines and reduce the tumor growth of xenograft models silenced for TSPAN1 and implanted in nude mice. Subsequently, it was interesting to discover that TSPAN1 could promote the process of autophagy both in vitro and in vivo through its interaction with the LC3 protein, thus promoting autophagosome maturation. In addition, it has been seen that the TSPAN1 expression was regulated by the WNT-CTNNB1 signaling pathway and correlated positively with the expression of the family with sequence similarity 83 member A (FAM83A), a protein associated with a poor prognosis of PC. Then, the authors identified 55 miRNAs differentially expressed in patients’ tissues, where 6 were upregulated and 49 were downregulated, with particular attention to miR454, which had the lowest expression levels in tumor tissues compared to healthy tissues. They further demonstrated that this miRNA was responsible for attenuating autophagy in cancerous cells by targeting TSPAN1 and by acting independently of mTOR and AMPK signaling pathways. Moreover, miR454 could also target the 3′-UTR region of FAM83A by negatively regulating the WNT-CTNNB1 pathway. So, these results showed that miR454 could play a tumor suppressor role in PC, whereas TSPAN1 and FAM83A could behave as promoters of neoplastic growth and progression.

On the other hand, Chen et al. [71] investigated the function of mir-372 in human pancreatic adenocarcinoma (HPAC). Initially, miR-372 expression was detected as downregulated in 20 pancreatic adenocarcinoma tissues compared to adjacent non-cancerous tissues, while LC3-II and p62 protein levels showed the opposite behavior. In fact, in vitro experiments highlighted that transfection with the miR-372 mimic vector in BXPC-3 and PANC-1 cells resulted in increased apoptosis and inhibition of cell migration and invasion by exerting its action on Unc-51-like autophagy activating kinase 1 (ULK1) as a direct target of miR-372, whose downregulation led to a decreased autophagy. Finally, ULK1 knockdown and chloroquine treatment are partially able to reverse the effects of miR-372 loss on HPAC cell viability, migration, and invasion, thus underlining the important role of miR-372 in tumor growth.

Finally, six independent studies that focused on the role of miRNAs in autophagy in PDAC are reported below.

Since it has been found that miR-7 acts as a tumor suppressor in common types of gastrointestinal cancers via regulating different signaling pathways, and in particular PI3K/Akt/mTOR axis [86,87,88,89,90], the aim of the work by Gu et al. [91] was to investigate whether miR-7 could destroy PC reprogrammed metabolic homeostasis through the regulation of an autophagy pathway and if it could affect cell proliferation and survival. They found that miR-7 overexpression blocked the glucose, or serum deprivation-induced autophagy in human PC cell lines via targeting autophagy-related key genes (i.e., ATG4A, ATG7, and ULK2). Furthermore, PC cell lines transfected with miR-7 mimics showed reduced cell proliferation, migration, and suppressed EMT, whilst inducing cell cycle arrest and apoptosis. Subsequently, they also assessed the biological effects of this miRNA in vivo, where a reduction of tumor growth and pancreatic tumor progression mediated by autophagy inhibition were observed in patient-derived xenografts (PDX) of mice.

Later, the same research group [92] investigated the expression levels of miR-7 in the serum of gemcitabine-resistance and gemcitabine-sensitive PC patients and healthy controls (HCs). Levels of miR-7 were significantly downregulated in PC patients compared to HCs and decreased in the gemcitabine-resistance group, with respect to the gemcitabine-sensitive. Following pancreatic tissue microarray-based validation, the authors noted that lower levels of miR-7 were strongly associated with poor prognosis and advanced stages of PC. Furthermore, they confirmed the previous findings about the crucial role of miR-7 in PC progression through the regulation of glycolytic metabolism via targeting autophagy.

Based on these findings, miR-7 could be a potential prognosis biomarker for patients affected by PC, and targeting miR-7 could represent a novel therapeutic approach for the treatment of PC patients.

Given the results obtained in previous works about the downregulation of miR-29 in PDAC, Kwon et al. [93] further investigated the possible molecular mechanism of this miRNA. After examining the expression of miR-29 in five PC cell lines (i.e., MIA PaCa-2, Panc-1, BxPC-3, COLO 357, and AsPC-1), they observed that four showed reduced expressions of both miR-29a and miR-29b, while miR-29c had a lower expression in only the three aforementioned cell lines compared to healthy pancreas cell lines. Principally, they focused their attention on the study of miR-29a, finding that miR-29a overexpressing cells treated with gemcitabine showed higher LDH production and caspase 3/7 activity with respect to cells treated with gemcitabine alone, thus supporting the idea that miR-29a increased the sensitivity of cancer cells to the drug treatment. Subsequently, transient transfection of the cell models with miR-29a led to an increase in LC3B protein and an accumulation of the autophagy substrate p62, similar to the results obtained with the use of CQ and bafilomycin A1 (BafA1), suggesting that miR-29a may play a role as a late-stage autophagy inhibitor. Furthermore, they observed that miR-29a may also block autophagic flux by preventing the fusion of the autophagosome with the lysosome. In fact, bioinformatic analysis and the luciferase reporter assay demonstrated that miR-29a was able to inhibit the autophagy process by targeting the autophagy-related gene 9A (ATG9A) and the transcription factor EB (TFEB), two pivotal molecules for autophagosome trafficking and lysosomal function, respectively. Finally, research about the role of miR-29a on migration, anchorage-independent growth, and the invasion of PC cells, has shown that this miRNA is also implicated in downregulating these cellular mechanisms.

In their work, Wu et al. [94] established that in cellular models of PDAC (i.e., PANC-1, CFP AC-1, PANC-198, and CAPAN-1) and in patients’ tissues, miR-9 played a role as a tumor repressor and sensitizer to chemotherapy treatment with doxorubicin; they attempted to explore the molecular mechanism by which this miRNA could act. Assuming a potential involvement of miR-9 in the doxorubicin-induced autophagy process, the expression levels of typical markers of this cellular process, including LC-3 I/II and p62, were evaluated. Firstly, they saw that miR-9 expression was inversely correlated with LC3 protein expression and autophagosome formation, and that this miRNA acted differently from CQ, but significantly increased Rapa-induced doxorubicin resistance, thus confirming its role in the autophagy process in PDAC cells. It could likely bind to the 3′-UTR region of eukaryotic translation initiation factor 5A2 (eIF5A2) mRNA, thereby inhibiting its production. Moreover, after devising a specific miR-9 delivery system consisting of nanoparticles selectively directed against the plectin-1 of PDACs (PL-1/miR-9 nanoparticles), the authors transfected these complexes in both in vitro and in vivo systems, finding an increased intracellular expression of miR-9 and a decreased eIF5A2 expression and autophagy activity, as well as increased sensitivity of tumor cells to doxorubicin treatment and cell apoptosis.

Sun et al. [95] investigated the correlation between the expression of MIR506 in PDAC and PC patients’ prognoses. They discovered that a low expression of MIR506 was associated with increased tumor progression and reduced patient survival. So, they performed further analyses to better understand the mechanisms of this miRNA in the pathogenesis of PDACs. Subsequent analyses showed that MIR506 was able to bind the 3′-UTR region of the signal transducer and activator of transcription 3 (STAT3) by regulating autophagic activity. Indeed, it has been observed that transfection of MIR506 into patient-derived xenograft cell lines resulted in a downregulation of STAT3 and BCL-2 expression and an increase of BECN1 levels and the autophagy process. Therefore, all of these results demonstrated that MIR506 could act as a tumor suppressor in PDACs by influencing the STAT3-BCL2-BECN1 axis and autophagy-related cell death.

Recently, a study conducted by Borchardt et al. [96] highlighted the possible oncosuppressor role of miR-24-3p in PDAC. Indeed, the miR-24 mimic transfection in four PDAC cell lines (i.e., Colo357, Panc89, PaTu-8988t, and Panc1) demonstrated that this miRNA led to a high reduction of cell viability with respect to negative control miR mimic and untransfected cells. Moreover, miR-24 overexpressing cells showed several changes in morphology and a reduction in the ability of colony formation and migration. In particular, the higher expression of miR-24 caused the downregulation of several factors, including MYC, survivin, Bax, Bad, Pim1, Pim2, and STAT3. On the other hand, there was an upregulation of the lipidated form of LC3 and autophagic flux, an increase of the levels of the cleaved form of poly (ADP-ribose)-polymerase 1 (PARP) and of p21 protein with the consequent reduction of the CDK6 function and cell progression in the cellular cycle. Furthermore, in vivo transfection with a specific nanoparticle/PEI/miR-24 mimic complex also led to a reduction in pancreatic tumor volume, and in agreement with the results obtained in vitro, this complex decreased the expression of p53, CDK6, survivin, and other proteins described above, while they observed an increase in the autophagic flux as well as the apoptotic and necrotic activities, confirming the tumor suppressor nature of miR-24.

In summary, all of these findings demonstrated that miRNAs play a fundamental role in PC development, progression, and resistance to drug treatment, showing their potential not only to become possible therapeutic targets but also as potential prognostic markers for this disease.

In Table 1, we summarize the differentially expressed autophagy-related miRNAs that have been suggested as potential biomarkers for pancreatic tumors.

## 3. Autophagy-Related lncRNAs in PC

In recent years, lncRNAs have attracted the attention of the scientific community for their involvement in crucial biological processes, including cell proliferation and differentiation, apoptosis, invasion, and metastasis, in patients affected by cancer [97,98]. Furthermore, emerging evidence indicated that their involvement in cancer occurrence and development could also be attributable to the effects of lncRNAs on autophagic flux [99,100]. In this light, in this section, we review studies that investigated autophagy-related lncRNAs in pancreatic tumors.

The lncRNA human plasmacytoma variant translocation 1 (PVT1) is an oncogenic lncRNA that has been reported to be involved in cancer progression and chemoresistance; however, mechanisms underlying its regulation in PC are not yet clear.

Similar work was carried out by Zhou et al. [101], who analyzed the possible mechanism by which PVT1 regulates the gemcitabine sensitivity of PC. They first observed that gemcitabine treatment induced (in a dose- and time-dependent manner) an upregulation of PVT1 in PC cells resistant to gemcitabine. Then, expression studies showed that PVT1 modulated Wnt/β-catenin signaling and autophagy activity by upregulating Pygo2 and ATG14 expression, thereby resulting in gemcitabine resistance of PC cell lines. Moreover, they observed that PVT1 increased the expressions of Pygo2 and ATG14 genes via sponging miR-619-5p. Finally, rescue assays exhibited that PVT1 interacted with ATG14, thereby promoting the autophagy-specific complex I (PtdIns3K-C1) assembly and ATG14-dependent class III PtdIns3K activity.

Finally, Liu et al. [102] evaluated the role of PVT1 in PC and whether its knockdown could suppress autophagy to enhance PC gemcitabine sensitivity via targeting the miR-143/HIF-1α/VMP1 signaling pathway. Quantitative real-time PCR (qPCR) results showed that levels of PVT1 were significantly increased while those of miR-143 were significantly reduced in carcinoma compared to the paracarcinoma tissues obtained from 36 PC patients. Cells knocked down for PVT1 or overexpressing miR-143 exhibited markedly-reduced autophagy activity and improved gemcitabine sensitivity. Functionally, they observed that this mechanism by which PVT1 regulated autophagy and gemcitabine sensitivity was through the modulation of the miR-143/HIF-1α/VMP1 axis.

Overall, these results support future research on PVT1 and autophagy for developing novel therapeutic strategies for treating human PC.

The purpose of the study performed by Liu et al. [74] was to obtain possible novel insight into the role of LINC01207 in apoptosis and autophagy and to clarify its regulatory mechanism in PC cells. Based on microarray and subsequent qPCR validation assays, the expression levels of LINC01207 and AGR2 were significantly upregulated, while those of miR-143-5p were decreased in PC tissues compared with the tumor-free cell-adjacent tissues. Mechanistically, LINC01207 was able to interact with and suppress miR-143-5p expression, which in turn targeted AGR2. LINC01207 silencing and miR-143-5p upregulation increased cell apoptosis and autophagy and their proteins, and in addition, decreased cell tumor growth. Therefore, intervening at this molecular level could prevent PC progression, thus providing novel molecular targets against PC.

Levels of lncRNA metastasis-associated lung adenocarcinoma transcript 1 (MALAT1) have been shown to increase in PC [103], suggesting MALAT1 as a potential diagnostic biomarker. Since the molecular mechanisms underlying the role of MALAT1, as well as tumor aggressiveness, remain poorly defined, Li et al. [104] investigated the clinical significance of the higher MALAT1 expression and autophagy in PDAC samples. MALAT1 expression levels were found to be increased and correlated with worse prognoses in PDAC tissue specimens. In vitro and in vivo experiments confirmed that the expressions of different autophagy-related molecules (i.e., LC3, P62, and LAMP-2) were modulated as a result of MALAT1 downregulation, leading to the inhibition of the autophagic process. Functionally, it was revealed that MALAT1 directly binds to RNA binding protein human antigen R (HuR), while no evidence supports its interaction with the tumor suppressor gene T-cell intracellular antigen-1 (TIA-1). Furthermore, MALAT1 downregulation inhibited the activation of autophagy by either suppressing HuR or by increasing the post-transcriptional regulation of TIA-1. Finally, they demonstrated that MALAT1 promoted PC proliferation and metastasis by enhancing autophagy both in vitro and in vivo. In summary, they concluded that lncRNA MALAT1 could serve as a potential predictive biomarker and therapeutic target for PC.

SNHG14 could function as an oncogene in cancer progression, even though gemcitabine resistance-associated mechanisms in PDAC remain poorly known. Zhang et al. [105] analyzed the PDAC-related tissue data obtained from The Cancer Genome Atlas (TCGA) database. This analysis revealed that the expression levels of SNHG14 were significantly higher, while those of miR-101 were significantly decreased in the PDAC tissues with respect to normal tissues, revealing an inversion correlation of gene expression between these molecules. The bioinformatic analysis and dual luciferase reporter assay indicated that SNHG14 functioned as a ceRNA by sponging miR-101 for subsequently promoting PDAC cell progression. In fact, increased expression of SNHG14 and the downregulation of miR-101 markedly promoted the proliferation, migration, and invasion of PDAC cells. Furthermore, an in vitro analysis revealed that SNHG14 knockdown or miR-101 upregulation significantly reduced PDAC cell resistance to gemcitabine and enhanced the apoptosis rate, as well as increased the levels of autophagy-related proteins, including RAB5A and ATG4D. In conclusion, they found that the SNHG14/miR-101/autophagy axis plays an important role in the chemosensitivity of gemcitabine in PDAC cells, providing new important findings for improving PDAC treatment.

Given the relevance of lncRNA ANRIL in supporting the development and progression of different tumors, Wang et al. [106] investigated the function of ANRIL in PC. qPCR and the Western blot analysis revealed that the levels of this lncRNA in PC were upregulated, as well as those of HMGB1, a predicted target gene of miR-181a [107], whose expression was significantly decreased in PC tissues compared with adjacent precancerous tissues. Such expression patterns were also found in PC cell lines, such as ASPC-1, PANC-1, HPAC, and BxPC-3, with respect to normal pancreatic cells, suggesting that miR-181a could be negatively regulated by ANRIL, resulting in increased HMGB1 expression. A subsequent luciferase reporter assay and in vitro experiments exhibited that miR-181a is able to directly target HMGB1 3′-UTR, thereby activating autophagic flux through upregulating the expression of autophagy-associated proteins, such as LC3 and BECN1. Furthermore, they observed that silencing of ANRIL in PC cell lines inhibited their proliferation, migration, and invasion, and reduced resistance to the gemcitabine-based chemotherapy, while the treatment with miR-181a reversed these effects. Collectively, these results suggest that ANRIL and miR-181a could be potential novel targets for the therapy of patients with PC.

An interesting bioinformatic study carried out by Tian et al. [108] aimed to identify and assess the feasibility of using an autophagy-associated lncRNA signature for predicting the survival of PC patients. Initially, the authors downloaded 182 lncRNAs from the TCGA website and 232 autophagy-associated genes from the Human Autophagy Database (HADb). Among these, 28 autophagy-related lncRNAs were identified by using the univariate Cox regression analysis for their potential as prognostic factors in PC.

Subsequently, a multivariate Cox regression analysis was carried out for optimizing the risk model, and 10 autophagy-related lncRNAs were included in the risk score tool for assessing PC patient prognosis. Results from this analysis revealed 10 autophagy-associated lncRNAs, including 4 predictors of poor prognosis (i.e., AC245041.2, AC036176.1, LINC01089, and LINC02257) and 6 positive prognosis factors (i.e., FLVCR1-DT, AC006504.7, AC125494.2, AC012306.2, ST20-AS1, and AC005696.1). The receiver operating characteristic (ROC) curve analysis demonstrated a good potential of the 10 selected lncRNAs for assessing clinical prognoses in PC patients, with an area under the curve (AUC) value of 0.815.

Although the research of novel biomarkers related to autophagy could be a promising avenue for PC patients, extensive research is needed to validate the differential expressions and elucidate the molecular mechanisms behind the role of autophagy-related lncRNAs in PC, to use these molecules for the development of novel targeted anticancer therapies and to help in the clinical assessment of PCs.

In Table 2, we summarize the differentially expressed autophagy-related lncRNAs that have been suggested as potential biomarkers for pancreatic tumors.

## 4. Autophagy-Related circRNAs in PC

Together with miRNAs and lncRNAs, circRNAs have emerged as a novel class of ncRNAs with different regulatory roles in various disorders [109]. For their key molecular functions, the aberrant expressions of circRNAs have been implicated in the progression of several diseases, including PC progression [110]. In this section, we will review two studies that investigate the relationship between specific circRNAs and autophagy in pancreatic tumors.

Yang et al. confirmed the previous results obtained from RNA sequencing analyses for identifying differentially expressed circRNAs between PDAC and adjacent normal tissues [111]. In this study [76], the authors characterized a circRNA derived from the *RHOBTB3* gene, named circRHOBTB3. Expression levels of this circRNA were significantly higher in PDAC tissues and human PDAC cell lines compared to their normal counterparts. Results from the loss and gain of function experiments showed that circRHOBTB3 stimulated PDAC proliferation both in vitro and in vivo by promoting the autophagic process through the suppression of Akt/mTOR in a mechanism that involves miR-600 and its target, nucleus accumbens associated 1 (NACC1). These findings suggest that a full understanding of the circRHOBTB3/miR-600/NACC1 axis could be a promising therapeutic strategy for PC.

Similar work was carried out by He et al. [75], who speculated that circRNA circ-autophagy related 7 (circATG7) could participate in PC progression by controlling ATG7. The authors found that the expression levels of circATG7 were upregulated in PC tissue with respect to normal adjacent normal tissue, and its levels positively correlated with lymph node metastasis and tumor diameters in PC patients.

The FISH analysis showed that circATG7 was located both in the nucleus and cytoplasm of PC cells and tissues. Moreover, they observed that overexpressed levels of this circRNA in the cytoplasm promote cell proliferation, autophagy, and migration in PC cells by sponging miR-766-5p, thus resulting in increased expression of ATG7, a target mRNA of this miRNA. Finally, confocal microscopy and an RNA-pull-down analysis revealed that nuclear circATG7 functioned as a scaffold to enhance the interaction between the HUR protein and ATG7 mRNA and increase the stability of the latter.

Consistent with these studies, these autophagy-related circRNAs could be novel potential biomarkers for PCs, even though the research is still in its infancy, and the underlying mechanisms of circRNAs in pancreatic tumor pathogenesis need further exploration.

In Table 3, we summarize the differentially expressed autophagy-related circRNAs that have been suggested as potential biomarkers for pancreatic tumors.

## 5. Discussion

The pancreas is one of the most important organs belonging to the endocrine system; it is mainly responsible for producing various hormones, including insulin and glucagon, and pancreatic enzymes that are involved in the digestion of nutrients. Dysfunctions occurring in this gland lead to the onset of various diseases, ranging from diabetes and pancreatitis to cancer. It has been estimated that the latter causes the death of three hundred thousand people per year, making it one of the leading cancer deaths [112].

Unfortunately, there are nonmodifiable risk factors (i.e., gender, age, ethnicity, blood group, and genetic factors) that predispose an individual to the development of PC. In fact, research shows that men are 30% more likely than women to be diagnosed with this disease [15]. In addition, ethnicity is considered an important factor in PC growth [15]. The incidence of the latter is higher in African Americans compared to Caucasians and Asians in the U.S. [113]. This is due to different lifestyles as well as the presence of gene mutations found more frequently in the former population than in the other two. In addition, several studies observed that some ethnic groups, such as Asians, precisely because of their genetic characteristics, show less aggressiveness to PC as well and have a higher survival rate. Moreover, ncRNAs, where expressions are regulated by lifestyle factors and interindividual differences, are involved in major or minor susceptibilities toward developing cancer [114,115]. Ke and colleagues [116] reported that single-nucleotide polymorphism (SNP) rs3802266 affecting miR-181a-2-3p binding to the 3′-UTR of the gene *ZHX2*, makes the Chinese population more prone to the occurrence of PC. On the contrary, the presence of the rs1859168 A > C SNP of lncRNA gene HOTTIP may decrease the PC risk in the Chinese population [117].

As far as surgical aspects, PC has been defined as resectable or unresectable, but in 2003, a new classification was introduced, defining the disease as ‘borderline resectable’ based on the relationship between the tumor and surrounding structures [118].

Undoubtedly, early diagnosis of PC remains crucial for the survival of patients, so they could be subjected to appropriate treatment plans in a timely manner, including the complete resection of the tumor, when possible, followed by the administration of chemotherapy. For patients who are not candidates for surgery, gemcitabine therapy remains the first-line option, sometimes administered in combination with other cytotoxic agents [119].

Unfortunately, many patients are prone to developing chemoresistance and do not respond to drug treatment; thus, they move toward disease progression [119].

Recent studies have proposed AdipoRon, a synthetic analog of adiponectin that acts on AdipoR1 and AdipoR2 receptors, as an anticancer drug to treat several types of cancer, especially PDACs [120]. In fact, it was reported that the use of AdipoRon on PC cells induced the latter to undergo a metabolic phenotype switch [121]. In addition, Ragone et al. [122] observed that the administration of this molecule in combination with gemcitabine led to a greater reduction of PC cell proliferation and the clonogenicity rate, as well as implemented susceptibility of chemoresistant cells to gemcitabine treatment. Certainly, it would be fascinating to understand more about the molecular mechanisms of this adiponectin mimic and whether it could potentially modulate the expressions of those ncRNAs involved in the pathogenesis of PCs in order to further promote AdipoRon entry into the clinic.

As some studies have demonstrated, the autophagic process is capable of playing a dual role in cancer, both as a tumor suppressor and promoter [123]. In most PC advanced stages, autophagy has been identified as one of the key processes for the survival and progression of cancer cells, even in a hostile microenvironment, allowing them to maintain metabolic homeostasis. This has made autophagy a promising target in the treatment of PC [40,41].

Autophagy is a highly conserved biological process that can be finely regulated by both genetic and epigenetic mechanisms. For instance, it is interesting to observe how the KRAS gene, which is frequently mutated in pancreatic tumors, is able to regulate the autophagy pathway via the activation of several signaling pathways [124].

In recent years, many studies on epigenetics have shown how these mechanisms may be involved in the onset, growth, and progression of several types of human cancers [125].

It is well known that ncRNAs, including circRNAs, lncRNAs, and miRNAs can indirectly or directly regulate gene expression through complex cross-talks between them [50].

Based on the findings reported in this review, the aberrant expressions of these molecules seem to be associated with the deregulation of autophagic flux and the pathogenesis of PC.

In addition, some of these ncRNAs described above have also been deregulated in other types of cancer. Indeed, altered expressions of miR-24, miR-506, and miR-7 appear to play important roles in the tumorigenesis of different cancers, including melanoma, glioma, and lung cancer [126,127,128].

In this review, the above-mentioned miRNAs were described as molecules with oncosuppressor behaviors, whose downregulation could lead to increased autophagic flux and, consequently, to tumor growth and progression, as well as resistance to drug treatment.

Moreover, the aberrant expressions of some lncRNAs mentioned in this review have also been shown to underlie the onset and pathogenesis of other cancers, such as MALAT1 and SNHG14, which seem to be possible diagnostic and prognostic therapeutic biomarkers in colorectal and hepatocellular cancer [129,130].

Due to the strong interest in ncRNAs as possible therapeutic targets, the scientific community has been engaged in developing innovative RNA-based therapeutic strategies. Among these, miRNA inhibitors and miRNA mimics are gaining the most ground in the biomedical field to negatively regulate the expressions of oncomiRNAs and positively regulate those of oncosuppressor miRNAs. Concerning lncRNA- and circRNA-based therapy—these are topics that are still in their infancy [131,132,133].

What is certain is that a therapy based on the use of ncRNAs could bring about great improvements to the life quality and clinical management of patients, as the use of these molecules as drugs could allow clinicians to develop targeted therapies for patients [50]. In addition, a greater selectivity of therapies could be provided by using magnetic nanoparticles complexed to ncRNAs to guide these molecules toward the diseased tissue by a magnetic field applied outside [134].

**Figure 1:**
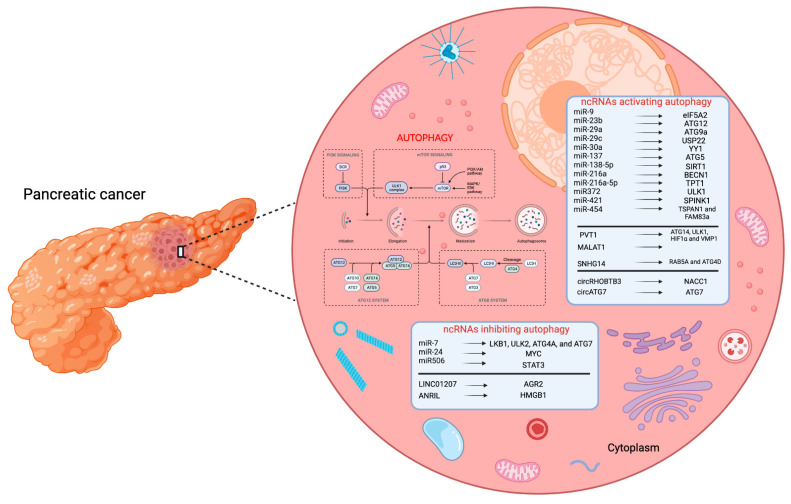
Overview of the autophagy-related ncRNAs that have been suggested as potential biomarkers in PC.

In conclusion, with this review, we offer an overview of the various miRNAs, lncRNAs, and circRNAs involved in tumorigenesis of PC (Figure 1), which could be considered as future diagnostic biomarkers to provide earlier diagnoses of this disease, as well as prognostic biomarkers to identify patients who respond (or not) to determined pharmacological treatments. Furthermore, these findings indicate that these molecules could serve as novel therapeutic targets for the development of ncRNA-based advanced therapies in PC.

## Figures and Tables

**Table 1:** Summary of differentially expressed autophagy-related miRNAs in PC.

microRNA	Expression Profile in PC	Impact on Autophagy	Autophagy-Related Targets	References
miR-154-3p andmiR-663a-5p	↓	N.D.	11 and 46	[77]
miR-216a-5p	↓	+	Translationally-controlled 1 (TPT1)	[78]
miR-23b	↓	+	Autophagy-related genes 12 (ATG12)	[79]
miR-216a	↓	+	Beclin 1 (BECN1)	[80]
miR-30a	↓	+	Yin-Yang 1 (YY1)	[81]
miR-138-5p	↓	+	Silent mating type information regulation 2 homolog 1 (SIRT1)	[82]
miR-29c	↓	+	Ubiquitin-specific peptidase 22 (USP22)	[72]
miR-137	↓	+	Autophagy-related genes 5 (ATG5)	[83]
miR-421	↓	+	Serine protease inhibitor Kazal-type 1 (SPINK1)	[84]
miR-454	↓	+	Tetraspanin 1 (TSPAN1) and FAM83A	[85]
miR-372	↓	+	Unc-51-like autophagy activating kinase 1 (ULK1)	[71]
miR-7	N.D.	−	Liver kinase B1 (LKB1), ULK2, ATG4A, and ATG7	[91]
miR-7	↓	−	N.D.	[92]
miR-29a	↓	−	Autophagy-related genes 9A (ATG9A)	[93]
miR-9	↓	+	Eukaryotic Translation Initiation Factor 5A2 (eIF5A2)	[94]
miR-506	↓	−	Signal transducer and activator of transcription 3 (STAT3)	[76]
miR-24	↓	−	MYC	[96]

N.D., data not determined; ↓, reduction in miRNA expression levels; +, activation of the autophagy signaling pathway; −, inhibition of autophagy signaling pathway.

**Table 2:** Summary of differentially expressed autophagy-related lncRNAs in PC.

lncRNA	Expression Profile in PC	Impact on Autophagy	Autophagy-Related Targets (miRNA)	References
PVT1	↑	+	ATG14 (miR-619-5p)	[101]
PVT1	↑	+	HIF-1α and VMP1 (miR-143)	[102]
LINC01207	↑	−	AGR2 (miR-143-5p)	[74]
MALAT1	↑	+	TIA-1	[104]
SNHG14	↑	+	RAB5A and ATG4D	[105]
ANRIL	↑	−	HMGB1 (miR-181a)	[106]
AC245041.2, AC036176.1, LINC01089, LINC02257, FLVCR1-DT, AC006504.7, AC125494.2, AC012306.2, ST20-AS1, and AC005696.1	N.D.	N.D.	N.D.	[108]

N.D., data not determined; ↑, increase in lncRNA expression levels; +, activation of autophagy signaling pathway; −, inhibition of autophagy signaling pathway.

**Table 3:** Summary of differentially expressed autophagy-related circRNAs in PC.

circRNA	Expression Profile in PC	Impact on Autophagy	Autophagy-Related Targets (miRNA)	References
circRHOBTB3	↑	+	NACC1 (miR-600)	[76]
circATG7	↑	+	ATG7 (miR-766-5p)	[75]

↑, increase in lncRNA expression levels; +, activation of the autophagy signaling pathway.

## Data Availability

Not applicable.

## References

[1] Röder P.V., Wu B., Liu Y., Han W. (2016). Pancreatic Regulation of Glucose Homeostasis. Exp. Mol. Med..

[2] Pan F.C., Wright C. (2011). Pancreas Organogenesis: From Bud to Plexus to Gland. Dev. Dyn..

[3] Gittes G.K. (2009). Developmental Biology of the Pancreas: A Comprehensive Review. Dev. Biol..

[4] Grant T.J., Hua K., Singh A. (2016). Molecular Pathogenesis of Pancreatic Cancer. Prog. Mol. Biol. Transl. Sci..

[5] Mahadevan V. (2019). Anatomy of the Pancreas and Spleen. Surg. Oxf..

[6] Polonsky K.S. (2012). The Past 200 Years in Diabetes. N. Engl. J. Med..

[7] Braganza J.M., Lee S.H., McCloy R.F., McMahon M.J. (2011). Chronic Pancreatitis. Lancet.

[8] Hezel A.F., Kimmelman A.C., Stanger B.Z., Bardeesy N., Depinho R.A. (2006). Genetics and Biology of Pancreatic Ductal Adenocarcinoma. Genes Dev..

[9] Klimstra D.S. (2007). Nonductal Neoplasms of the Pancreas. Mod. Pathol. Off. J. U.S. Can. Acad. Pathol. Inc..

[10] Luchini C., Grillo F., Fassan M., Vanoli A., Capelli P., Paolino G., Ingravallo G., Renzulli G., Doglioni C., D’Amuri A. (2020). Malignant Epithelial/Exocrine Tumors of the Pancreas. Pathologica.

[11] Sridharan V., Hernandez-Barco Y.G., Ting D.T. (2020). Landscape of Circulating Diagnostic Biomarkers in Pancreatic Malignancies. Ann. Pancreat. Cancer.

[12] Kanji Z.S., Gallinger S. (2013). Diagnosis and Management of Pancreatic Cancer. CMAJ Can. Med. Assoc. J. J. Assoc. Medicale Can..

[13] Costache M.I., Costache C.A., Dumitrescu C.I., Tica A.A., Popescu M., Baluta E.A., Anghel A.C., Saftoiu A., Dumitrescu D. (2017). Which Is the Best Imaging Method in Pancreatic Adenocarcinoma Diagnosis and Staging—CT, MRI or EUS?. Curr. Health Sci. J..

[14] Pekarek L., Fraile-Martinez O., Garcia-Montero C., Saez M.A., Barquero-Pozanco I., Del Hierro-Marlasca L., de Castro Martinez P., Romero-Bazán A., Alvarez-Mon M.A., Monserrat J. (2022). Clinical Applications of Classical and Novel Biological Markers of Pancreatic Cancer. Cancers.

[15] Wörmann S.M., Algül H. (2013). Risk Factors and Therapeutic Targets in Pancreatic Cancer. Front. Oncol..

[16] Risch H.A., Lu L., Kidd M.S., Wang J., Zhang W., Ni Q., Gao Y.-T., Yu H. (2014). Helicobacter Pylori Seropositivities and Risk of Pancreatic Carcinoma. Cancer Epidemiol. Biomark. Prev. Publ. Am. Assoc. Cancer Res. Cosponsored Am. Soc. Prev. Oncol..

[17] Hassan M.M., Li D., El-Deeb A.S., Wolff R.A., Bondy M.L., Davila M., Abbruzzese J.L. (2008). Association Between Hepatitis B Virus and Pancreatic Cancer. J. Clin. Oncol..

[18] El-Serag H.B., Engels E.A., Landgren O., Chiao E., Henderson L., Amaratunge H.C., Giordano T.P. (2009). Risk of Hepatobiliary and Pancreatic Cancers after Hepatitis C Virus Infection: A Population-Based Study of U.S. Veterans. Hepatology.

[19] Artinyan A., Soriano P.A., Prendergast C., Low T., Ellenhorn J.D.I., Kim J. (2008). The Anatomic Location of Pancreatic Cancer Is a Prognostic Factor for Survival. HPB.

[20] Baur A.D.J., Pavel M., Prasad V., Denecke T. (2016). Diagnostic Imaging of Pancreatic Neuroendocrine Neoplasms (PNEN): Tumor Detection, Staging, Prognosis, and Response to Treatment. Acta Radiol..

[21] Viúdez A., De Jesus-Acosta A., Carvalho F.L., Vera R., Martín-Algarra S., Ramírez N. (2016). Pancreatic Neuroendocrine Tumors: Challenges in an Underestimated Disease. Crit. Rev. Oncol. Hematol..

[22] Stathis A., Moore M.J. (2010). Advanced Pancreatic Carcinoma: Current Treatment and Future Challenges. Nat. Rev. Clin. Oncol..

[23] Seufferlein T., Bachet J.B., Van Cutsem E., Rougier P., ESMO Guidelines Working Group (2012). Pancreatic Adenocarcinoma: ESMO-ESDO Clinical Practice Guidelines for Diagnosis, Treatment and Follow-Up. Ann. Oncol. Off. J. Eur. Soc. Med. Oncol..

[24] Zhang Q., Zeng L., Chen Y., Lian G., Qian C., Chen S., Li J., Huang K. (2016). Pancreatic Cancer Epidemiology, Detection, and Management. Gastroenterol. Res. Pract..

[25] D’Cruz J.R., Misra S., Shamsudeen S. (2022). Pancreaticoduodenectomy. StatPearls.

[26] Edwards P., Kang B.W., Chau I. (2021). Targeting the Stroma in the Management of Pancreatic Cancer. Front. Oncol..

[27] Zhu H., Li T., Du Y., Li M. (2018). Pancreatic Cancer: Challenges and Opportunities. BMC Med..

[28] Kelly P.N. (2018). The Cancer Immunotherapy Revolution. Science.

[29] Yoon J.H., Jung Y.-J., Moon S.-H. (2021). Immunotherapy for Pancreatic Cancer. World J. Clin. Cases.

[30] Adamska A., Domenichini A., Falasca M. (2017). Pancreatic Ductal Adenocarcinoma: Current and Evolving Therapies. Int. J. Mol. Sci..

[31] Sohal D.P.S., Kennedy E.B., Cinar P., Conroy T., Copur M.S., Crane C.H., Garrido-Laguna I., Lau M.W., Johnson T., Krishnamurthi S. (2020). Metastatic Pancreatic Cancer: ASCO Guideline Update. J. Clin. Oncol. Off. J. Am. Soc. Clin. Oncol..

[32] Humphris J.L., Patch A.-M., Nones K., Bailey P.J., Johns A.L., McKay S., Chang D.K., Miller D.K., Pajic M., Kassahn K.S. (2017). Hypermutation In Pancreatic Cancer. Gastroenterology.

[33] Nevala-Plagemann C., Hidalgo M., Garrido-Laguna I. (2020). From State-of-the-Art Treatments to Novel Therapies for Advanced-Stage Pancreatic Cancer. Nat. Rev. Clin. Oncol..

[34] Mucciolo G., Roux C., Scagliotti A., Brugiapaglia S., Novelli F., Cappello P. (2020). The Dark Side of Immunotherapy: Pancreatic Cancer. Cancer Drug Resist..

[35] Mizushima N., Kuma A., Kobayashi Y., Yamamoto A., Matsubae M., Takao T., Natsume T., Ohsumi Y., Yoshimori T. (2003). Mouse Apg16L, a Novel WD-Repeat Protein, Targets to the Autophagic Isolation Membrane with the Apg12-Apg5 Conjugate. J. Cell Sci..

[36] Perrotta C., Cattaneo M.G., Molteni R., De Palma C. (2020). Autophagy in the Regulation of Tissue Differentiation and Homeostasis. Front. Cell Dev. Biol..

[37] Mathiassen S.G., De Zio D., Cecconi F. (2017). Autophagy and the Cell Cycle: A Complex Landscape. Front. Oncol..

[38] Klionsky D.J. (2007). Autophagy: From Phenomenology to Molecular Understanding in Less than a Decade. Nat. Rev. Mol. Cell Biol..

[39] Cao W., Li J., Yang K., Cao D. (2021). An Overview of Autophagy: Mechanism, Regulation and Research Progress. Bull. Cancer.

[40] Yang M.-C., Wang H.-C., Hou Y.-C., Tung H.-L., Chiu T.-J., Shan Y.-S. (2015). Blockade of Autophagy Reduces Pancreatic Cancer Stem Cell Activity and Potentiates the Tumoricidal Effect of Gemcitabine. Mol. Cancer.

[41] Yang S., Wang X., Contino G., Liesa M., Sahin E., Ying H., Bause A., Li Y., Stommel J.M., Dell’antonio G. (2011). Pancreatic Cancers Require Autophagy for Tumor Growth. Genes Dev..

[42] Li J., Chen X., Kang R., Zeh H., Klionsky D.J., Tang D. (2021). Regulation and Function of Autophagy in Pancreatic Cancer. Autophagy.

[43] Hwang R.F., Moore T., Arumugam T., Ramachandran V., Amos K.D., Rivera A., Ji B., Evans D.B., Logsdon C.D. (2008). Cancer-Associated Stromal Fibroblasts Promote Pancreatic Tumor Progression. Cancer Res..

[44] Endo S., Nakata K., Ohuchida K., Takesue S., Nakayama H., Abe T., Koikawa K., Okumura T., Sada M., Horioka K. (2017). Autophagy Is Required for Activation of Pancreatic Stellate Cells, Associated with Pancreatic Cancer Progression and Promotes Growth of Pancreatic Tumors in Mice. Gastroenterology.

[45] Yang A., Kimmelman A.C. (2014). Inhibition of Autophagy Attenuates Pancreatic Cancer Growth Independent of TP53/TRP53 Status. Autophagy.

[46] Sousa C.M., Biancur D.E., Wang X., Halbrook C.J., Sherman M.H., Zhang L., Kremer D., Hwang R.F., Witkiewicz A.K., Ying H. (2016). Pancreatic Stellate Cells Support Tumour Metabolism through Autophagic Alanine Secretion. Nature.

[47] Pipinikas C.P., Berner A.M., Sposito T., Thirlwell C. (2019). The Evolving (Epi)Genetic Landscape of Pancreatic Neuroendocrine Tumours. Endocr. Relat. Cancer.

[48] Marini F., Cianferotti L., Brandi M.L. (2016). Epigenetic Mechanisms in Bone Biology and Osteoporosis: Can They Drive Therapeutic Choices?. Int. J. Mol. Sci..

[49] Aurilia C., Donati S., Palmini G., Miglietta F., Iantomasi T., Brandi M.L. (2021). The Involvement of Long Non-Coding RNAs in Bone. Int. J. Mol. Sci..

[50] Aurilia C., Donati S., Palmini G., Miglietta F., Falsetti I., Iantomasi T., Brandi M.L. (2021). Are Non-Coding RNAs Useful Biomarkers in Parathyroid Tumorigenesis?. Int. J. Mol. Sci..

[51] Marques A.C., Hughes J., Graham B., Kowalczyk M.S., Higgs D.R., Ponting C.P. (2013). Chromatin Signatures at Transcriptional Start Sites Separate Two Equally Populated yet Distinct Classes of Intergenic Long Noncoding RNAs. Genome Biol..

[52] Salzman J., Gawad C., Wang P.L., Lacayo N., Brown P.O. (2012). Circular RNAs Are the Predominant Transcript Isoform from Hundreds of Human Genes in Diverse Cell Types. PLoS ONE.

[53] Wang K.C., Chang H.Y. (2011). Molecular Mechanisms of Long Noncoding RNAs. Mol. Cell.

[54] Guo Q., Guo Q., Xiao Y., Li C., Huang Y., Luo X. (2020). Regulation of Bone Marrow Mesenchymal Stem Cell Fate by Long Non-Coding RNA. Bone.

[55] Wilusz J.E. (2018). A 360° View of Circular RNAs: From Biogenesis to Functions. Wiley Interdiscip. Rev. RNA.

[56] Carthew R.W., Sontheimer E.J. (2009). Origins and Mechanisms of MiRNAs and SiRNAs. Cell.

[57] Flynt A.S., Lai E.C. (2008). Biological Principles of MicroRNA-Mediated Regulation: Shared Themes amid Diversity. Nat. Rev. Genet..

[58] Lee R.C., Feinbaum R.L., Ambros V. (1993). The C. Elegans Heterochronic Gene Lin-4 Encodes Small RNAs with Antisense Complementarity to Lin-14. Cell.

[59] Wightman B., Ha I., Ruvkun G. (1993). Posttranscriptional Regulation of the Heterochronic Gene Lin-14 by Lin-4 Mediates Temporal Pattern Formation in C. Elegans. Cell.

[60] Hsu P.W.C., Huang H.-D., Hsu S.-D., Lin L.-Z., Tsou A.-P., Tseng C.-P., Stadler P.F., Washietl S., Hofacker I.L. (2006). MiRNAMap: Genomic Maps of MicroRNA Genes and Their Target Genes in Mammalian Genomes. Nucleic Acids Res..

[61] Malan-Müller S., Hemmings S.M.J., Seedat S. (2013). Big Effects of Small RNAs: A Review of MicroRNAs in Anxiety. Mol. Neurobiol..

[62] Huntzinger E., Izaurralde E. (2011). Gene Silencing by MicroRNAs: Contributions of Translational Repression and MRNA Decay. Nat. Rev. Genet..

[63] Ipsaro J.J., Joshua-Tor L. (2015). From Guide to Target: Molecular Insights into Eukaryotic RNA-Interference Machinery. Nat. Struct. Mol. Biol..

[64] Donati S., Ciuffi S., Brandi M.L. (2019). Human Circulating MiRNAs Real-Time QRT-PCR-Based Analysis: An Overview of Endogenous Reference Genes Used for Data Normalization. Int. J. Mol. Sci..

[65] O’Brien J., Hayder H., Zayed Y., Peng C. (2018). Overview of MicroRNA Biogenesis, Mechanisms of Actions, and Circulation. Front. Endocrinol..

[66] Liang S., Li X., Gao C., Zhang L. (2020). MicroRNA-Based Autophagy Inhibition as Targeted Therapy in Pancreatic Cancer. Biomed. Pharmacother..

[67] Mortoglou M., Tabin Z.K., Arisan E.D., Kocher H.M., Uysal-Onganer P. (2021). Non-Coding RNAs in Pancreatic Ductal Adenocarcinoma: New Approaches for Better Diagnosis and Therapy. Transl. Oncol..

[68] Esmaeili M., Keshani M., Vakilian M., Esmaeili M., Peymani M., Seyed Forootan F., Chau T.L., Göktuna S.I., Zaker S.R., Nasr Esfahani M.H. (2020). Role of Non-Coding RNAs as Novel Biomarkers for Detection of Colorectal Cancer Progression through Interaction with the Cell Signaling Pathways. Gene.

[69] Zhu H., Wu H., Liu X., Li B., Chen Y., Ren X., Liu C.-G., Yang J.-M. (2009). Regulation of Autophagy by a Beclin 1-Targeted MicroRNA, MiR-30a, in Cancer Cells. Autophagy.

[70] Chen W., Zhou Y., Zhi X., Ma T., Liu H., Chen B.W., Zheng X., Xie S., Zhao B., Feng X. (2019). Delivery of MiR-212 by Chimeric Peptide-Condensed Supramolecular Nanoparticles Enhances the Sensitivity of Pancreatic Ductal Adenocarcinoma to Doxorubicin. Biomaterials.

[71] Chen H., Zhang Z., Lu Y., Song K., Liu X., Xia F., Sun W. (2017). Downregulation of ULK1 by MicroRNA-372 Inhibits the Survival of Human Pancreatic Adenocarcinoma Cells. Cancer Sci..

[72] Huang L., Hu C., Cao H., Wu X., Wang R., Lu H., Li H., Chen H. (2018). MicroRNA-29c Increases the Chemosensitivity of Pancreatic Cancer Cells by Inhibiting USP22 Mediated Autophagy. Cell. Physiol. Biochem. Int. J. Exp. Cell. Physiol. Biochem. Pharmacol..

[73] Pang E.-J., Yang R., Fu X., Liu Y. (2015). Overexpression of Long Non-Coding RNA MALAT1 Is Correlated with Clinical Progression and Unfavorable Prognosis in Pancreatic Cancer. Tumour Biol. J. Int. Soc. Oncodevelopmental Biol. Med..

[74] Liu C., Wang J.-O., Zhou W.-Y., Chang X.-Y., Zhang M.-M., Zhang Y., Yang X.-H. (2019). Long Non-Coding RNA LINC01207 Silencing Suppresses AGR2 Expression to Facilitate Autophagy and Apoptosis of Pancreatic Cancer Cells by Sponging MiR-143-5p. Mol. Cell. Endocrinol..

[75] He Z., Cai K., Zeng Z., Lei S., Cao W., Li X. (2022). Autophagy-Associated CircRNA CircATG7 Facilitates Autophagy and Promotes Pancreatic Cancer Progression. Cell Death Dis..

[76] Yang T., Shen P., Chen Q., Wu P., Yuan H., Ge W., Meng L., Huang X., Fu Y., Zhang Y. (2021). FUS-Induced CircRHOBTB3 Facilitates Cell Proliferation via MiR-600/NACC1 Mediated Autophagy Response in Pancreatic Ductal Adenocarcinoma. J. Exp. Clin. Cancer Res..

[77] Wei D.-M., Jiang M.-T., Lin P., Yang H., Dang Y.-W., Yu Q., Liao D.-Y., Luo D.-Z., Chen G. (2018). Potential CeRNA Networks Involved in Autophagy Suppression of Pancreatic Cancer Caused by Chloroquine Diphosphate: A Study Based on Differentially-Expressed CircRNAs, LncRNAs, MiRNAs and MRNAs. Int. J. Oncol..

[78] Zhang J., Gao S., Zhang Y., Yi H., Xu M., Xu J., Liu H., Ding Z., He H., Wang H. (2020). MiR-216a-5p Inhibits Tumorigenesis in Pancreatic Cancer by Targeting TPT1/MTORC1 and Is Mediated by LINC01133. Int. J. Biol. Sci..

[79] Wang P., Zhang J., Zhang L., Zhu Z., Fan J., Chen L., Zhuang L., Luo J., Chen H., Liu L. (2013). MicroRNA 23b Regulates Autophagy Associated with Radioresistance of Pancreatic Cancer Cells. Gastroenterology.

[80] Zhang X., Shi H., Lin S., Ba M., Cui S. (2015). MicroRNA-216a Enhances the Radiosensitivity of Pancreatic Cancer Cells by Inhibiting Beclin-1-Mediated Autophagy. Oncol. Rep..

[81] Yang C., Zhang J.-J., Peng Y.-P., Zhu Y., Yin L.-D., Wei J.-S., Gao W.-T., Jiang K.-R., Miao Y. (2017). A Yin-Yang 1/MiR-30a Regulatory Circuit Modulates Autophagy in Pancreatic Cancer Cells. J. Transl. Med..

[82] Tian S., Guo X., Yu C., Sun C., Jiang J. (2016). MiR-138-5p Suppresses Autophagy in Pancreatic Cancer by Targeting SIRT1. Oncotarget.

[83] Wang Z.-C., Huang F.-Z., Xu H.-B., Sun J.-C., Wang C.-F. (2019). MicroRNA-137 Inhibits Autophagy and Chemosensitizes Pancreatic Cancer Cells by Targeting ATG5. Int. J. Biochem. Cell Biol..

[84] Shopit A., Li X., Tang Z., Awsh M., Shobet L., Niu M., Wang H., Mousa H., Alshwmi M., Tesfaldet T. (2020). MiR-421 up-Regulation by the Oleanolic Acid Derivative K73-03 Regulates Epigenetically SPINK1 Transcription in Pancreatic Cancer Cells Leading to Metabolic Changes and Enhanced Apoptosis. Pharmacol. Res..

[85] Zhou C., Liang Y., Zhou L., Yan Y., Liu N., Zhang R., Huang Y., Wang M., Tang Y., Ali D.W. (2021). TSPAN1 Promotes Autophagy Flux and Mediates Cooperation between WNT-CTNNB1 Signaling and Autophagy via the MIR454-FAM83A-TSPAN1 Axis in Pancreatic Cancer. Autophagy.

[86] Xie J., Chen M., Zhou J., Mo M.-S., Zhu L.-H., Liu Y.-P., Gui Q.-J., Zhang L., Li G.-Q. (2014). MiR-7 Inhibits the Invasion and Metastasis of Gastric Cancer Cells by Suppressing Epidermal Growth Factor Receptor Expression. Oncol. Rep..

[87] Fang Y., Xue J.-L., Shen Q., Chen J., Tian L. (2012). MicroRNA-7 Inhibits Tumor Growth and Metastasis by Targeting the Phosphoinositide 3-Kinase/Akt Pathway in Hepatocellular Carcinoma. Hepatology.

[88] Gu D.-N., Huang Q., Tian L. (2015). The Molecular Mechanisms and Therapeutic Potential of MicroRNA-7 in Cancer. Expert Opin. Ther. Targets.

[89] Zhang N., Li X., Wu C.W., Dong Y., Cai M., Mok M.T.S., Wang H., Chen J., Ng S.S.M., Chen M. (2013). MicroRNA-7 Is a Novel Inhibitor of YY1 Contributing to Colorectal Tumorigenesis. Oncogene.

[90] Zhao X.-D., Lu Y.-Y., Guo H., Xie H.-H., He L.-J., Shen G.-F., Zhou J.-F., Li T., Hu S.-J., Zhou L. (2015). MicroRNA-7/NF-ΚB Signaling Regulatory Feedback Circuit Regulates Gastric Carcinogenesis. J. Cell Biol..

[91] Gu D.-N., Jiang M.-J., Mei Z., Dai J.-J., Dai C.-Y., Fang C., Huang Q., Tian L. (2017). MicroRNA-7 Impairs Autophagy-Derived Pools of Glucose to Suppress Pancreatic Cancer Progression. Cancer Lett..

[92] Ye Z., Zou C., Chen H., Jiang M., Mei Z., Gu D. (2020). MicroRNA-7 as a Potential Biomarker for Prognosis in Pancreatic Cancer. Dis. Markers.

[93] Kwon J.J., Willy J.A., Quirin K.A., Wek R.C., Korc M., Yin X.-M., Kota J. (2016). Novel Role of MiR-29a in Pancreatic Cancer Autophagy and Its Therapeutic Potential. Oncotarget.

[94] Wu Y., Tang Y., Xie S., Zheng X., Zhang S., Mao J., Wang B., Hou Y., Hu L., Chai K. (2020). Chimeric Peptide Supramolecular Nanoparticles for Plectin-1 Targeted MiRNA-9 Delivery in Pancreatic Cancer. Theranostics.

[95] Sun L., Hu L., Cogdell D., Lu L., Gao C., Tian W., Zhang Z., Kang Y., Fleming J.B., Zhang W. (2017). MIR506 Induces Autophagy-Related Cell Death in Pancreatic Cancer Cells by Targeting the STAT3 Pathway. Autophagy.

[96] Borchardt H., Ewe A., Morawski M., Weirauch U., Aigner A. (2021). MiR24-3p Activity after Delivery into Pancreatic Carcinoma Cell Lines Exerts Profound Tumor-Inhibitory Effects through Distinct Pathways of Apoptosis and Autophagy Induction. Cancer Lett..

[97] Bermúdez M., Aguilar-Medina M., Lizárraga-Verdugo E., Avendaño-Félix M., Silva-Benítez E., López-Camarillo C., Ramos-Payán R. (2019). LncRNAs as Regulators of Autophagy and Drug Resistance in Colorectal Cancer. Front. Oncol..

[98] Xu X., Cui L., Zhong W., Cai Y. (2020). Autophagy-Associated LncRNAs: Promising Targets for Neurological Disease Diagnosis and Therapy. Neural Plast..

[99] Li X., Jin F., Li Y. (2021). A Novel Autophagy-Related LncRNA Prognostic Risk Model for Breast Cancer. J. Cell. Mol. Med..

[100] Yang L., Wang H., Shen Q., Feng L., Jin H. (2017). Long Non-Coding RNAs Involved in Autophagy Regulation. Cell Death Dis..

[101] Zhou C., Yi C., Yi Y., Qin W., Yan Y., Dong X., Zhang X., Huang Y., Zhang R., Wei J. (2020). LncRNA PVT1 Promotes Gemcitabine Resistance of Pancreatic Cancer via Activating Wnt/β-Catenin and Autophagy Pathway through Modulating the MiR-619-5p/Pygo2 and MiR-619-5p/ATG14 Axes. Mol. Cancer.

[102] Liu Y.-F., Luo D., Li X., Li Z.-Q., Yu X., Zhu H.-W. (2021). PVT1 Knockdown Inhibits Autophagy and Improves Gemcitabine Sensitivity by Regulating the MiR-143/HIF-1α/VMP1 Axis in Pancreatic Cancer. Pancreas.

[103] Jiao F., Hu H., Yuan C., Wang L., Jiang W., Jin Z., Guo Z., Wang L. (2014). Elevated Expression Level of Long Noncoding RNA MALAT-1 Facilitates Cell Growth, Migration and Invasion in Pancreatic Cancer. Oncol. Rep..

[104] Li L., Chen H., Gao Y., Wang Y.-W., Zhang G.-Q., Pan S.-H., Ji L., Kong R., Wang G., Jia Y.-H. (2016). Long Noncoding RNA MALAT1 Promotes Aggressive Pancreatic Cancer Proliferation and Metastasis via the Stimulation of Autophagy. Mol. Cancer Ther..

[105] Zhang X., Zhao P., Wang C., Xin B. (2019). SNHG14 Enhances Gemcitabine Resistance by Sponging MiR-101 to Stimulate Cell Autophagy in Pancreatic Cancer. Biochem. Biophys. Res. Commun..

[106] Wang L., Bi R., Li L., Zhou K., Yin H. (2021). LncRNA ANRIL Aggravates the Chemoresistance of Pancreatic Cancer Cells to Gemcitabine by Targeting Inhibition of MiR-181a and Targeting HMGB1-Induced Autophagy. Aging.

[107] Dahlhaus M., Schult C., Lange S., Freund M., Junghanss C. (2013). MicroRNA 181a Influences the Expression of HMGB1 and CD4 in Acute Leukemias. Anticancer Res..

[108] Tian J., Fu C., Zeng X., Fan X., Wu Y. (2022). An Independent Prognostic Model Based on Ten Autophagy-Related Long Noncoding RNAs in Pancreatic Cancer Patients. Genet. Res..

[109] Hsiao K.-Y., Sun H.S., Tsai S.-J. (2017). Circular RNA—New Member of Noncoding RNA with Novel Functions. Exp. Biol. Med..

[110] Rong Z., Xu J., Shi S., Tan Z., Meng Q., Hua J., Liu J., Zhang B., Wang W., Yu X. (2021). Circular RNA in Pancreatic Cancer: A Novel Avenue for the Roles of Diagnosis and Treatment. Theranostics.

[111] Shen P., Yang T., Chen Q., Yuan H., Wu P., Cai B., Meng L., Huang X., Liu J., Zhang Y. (2021). CircNEIL3 Regulatory Loop Promotes Pancreatic Ductal Adenocarcinoma Progression via MiRNA Sponging and A-to-I RNA-Editing. Mol. Cancer.

[112] Ferlay J., Soerjomataram I., Dikshit R., Eser S., Mathers C., Rebelo M., Parkin D.M., Forman D., Bray F. (2015). Cancer Incidence and Mortality Worldwide: Sources, Methods and Major Patterns in GLOBOCAN 2012. Int. J. Cancer.

[113] Midha S., Chawla S., Garg P.K. (2016). Modifiable and Non-Modifiable Risk Factors for Pancreatic Cancer: A Review. Cancer Lett..

[114] Panico A., Tumolo M.R., Leo C.G., Donno A.D., Grassi T., Bagordo F., Serio F., Idolo A., Masi R.D., Mincarone P. (2021). The Influence of Lifestyle Factors on MiRNA Expression and Signal Pathways: A Review. Epigenomics.

[115] Gong Z., Wang J., Wang D., Buas M.F., Ren X., Freudenheim J.L., Belinsky S.A., Liu S., Ambrosone C.B., Higgins M.J. (2019). Differences in MicroRNA Expression in Breast Cancer between Women of African and European Ancestry. Carcinogenesis.

[116] Ke J., Peng X., Mei S., Tian J., Ying P., Yang N., Wang X., Zou D., Yang Y., Zhu Y. (2020). Evaluation of Polymorphisms in MicroRNA-Binding Sites and Pancreatic Cancer Risk in Chinese Population. J. Cell. Mol. Med..

[117] Hu P., Qiao O., Wang J., Li J., Jin H., Li Z., Jin Y. (2017). Rs1859168 A > C Polymorphism Regulates HOTTIP Expression and Reduces Risk of Pancreatic Cancer in a Chinese Population. World J. Surg. Oncol..

[118] Mohammed S., Van Buren G., Fisher W.E. (2014). Pancreatic Cancer: Advances in Treatment. World J. Gastroenterol..

[119] Ansari D., Gustafsson A., Andersson R. (2015). Update on the Management of Pancreatic Cancer: Surgery Is Not Enough. World J. Gastroenterol..

[120] Sapio L., Ragone A., Spina A., Salzillo A., Naviglio S. (2022). AdipoRon and Pancreatic Ductal Adenocarcinoma: A Future Perspective in Overcoming Chemotherapy-Induced Resistance?. Cancer Drug Resist..

[121] Manley S.J., Olou A.A., Jack J.L., Ruckert M.T., Walsh R.M., Eades A.E., Bye B.A., Ambrose J., Messaggio F., Anant S. (2022). Synthetic Adiponectin-Receptor Agonist, AdipoRon, Induces Glycolytic Dependence in Pancreatic Cancer Cells. Cell Death Dis..

[122] Ragone A., Salzillo A., Spina A., Naviglio S., Sapio L. (2022). Integrating Gemcitabine-Based Therapy With AdipoRon Enhances Growth Inhibition in Human PDAC Cell Lines. Front. Pharmacol..

[123] White E. (2015). The Role for Autophagy in Cancer. J. Clin. Investig..

[124] Poillet-Perez L., Despouy G., Delage-Mourroux R., Boyer-Guittaut M. (2015). Interplay between ROS and Autophagy in Cancer Cells, from Tumor Initiation to Cancer Therapy. Redox Biol..

[125] Hackl M., Heilmeier U., Weilner S., Grillari J. (2016). Circulating MicroRNAs as Novel Biomarkers for Bone Diseases—Complex Signatures for Multifactorial Diseases?. Mol. Cell. Endocrinol..

[126] Li J., Ju J., Ni B., Wang H. (2016). The Emerging Role of MiR-506 in Cancer. Oncotarget.

[127] Mukherjee S., Shelar B., Krishna S. (2022). Versatile Role of MiR-24/24-1*/24-2* Expression in Cancer and Other Human Diseases. Am. J. Transl. Res..

[128] Korać P., Antica M., Matulić M. (2021). MiR-7 in Cancer Development. Biomedicines.

[129] Shen S., Wang Y., Zhang Y., Dong Z., Xing J. (2021). Long Non-Coding RNA Small Nucleolar RNA Host Gene 14, a Promising Biomarker and Therapeutic Target in Malignancy. Front. Cell Dev. Biol..

[130] Ghafouri-Fard S., Ashrafi Hafez A., Taheri M. (2020). Metastasis Associated Lung Adenocarcinoma Transcript 1: An Update on Expression Pattern and Functions in Carcinogenesis. Exp. Mol. Pathol..

[131] Bajan S., Hutvagner G. (2020). RNA-Based Therapeutics: From Antisense Oligonucleotides to MiRNAs. Cells.

[132] Ling H., Fabbri M., Calin G.A. (2013). MicroRNAs and Other Non-Coding RNAs as Targets for Anticancer Drug Development. Nat. Rev. Drug Discov..

[133] Winkle M., El-Daly S.M., Fabbri M., Calin G.A. (2021). Noncoding RNA Therapeutics—Challenges and Potential Solutions. Nat. Rev. Drug Discov..

[134] Tietze R., Zaloga J., Unterweger H., Lyer S., Friedrich R.P., Janko C., Pöttler M., Dürr S., Alexiou C. (2015). Magnetic Nanoparticle-Based Drug Delivery for Cancer Therapy. Biochem. Biophys. Res. Commun..

